# Case report: Rare isolated cystic hepatic metastasis of a patient with squamous cell lung carcinoma history and the prognosis

**DOI:** 10.3389/fonc.2022.986603

**Published:** 2022-10-27

**Authors:** Chunbao Liu, Xiaomin Chen, Hang Su, Liang Xia, Diyu Lu

**Affiliations:** ^1^ Department of Nuclear Medicine, The Central Hospital of Wuhan, Tongji Medical College, Huazhong University of Science and Technology, Wuhan, China; ^2^ Key Laboratory for Molecular Diagnosis of Hubei Province, The Central Hospital of Wuhan, Tongji Medical College, Huazhong University of Science and Technology, Wuhan, China

**Keywords:** cystic metastasis, squamous cell lung carcinoma, PET/CT, immunotherapy, 18F-FDG

## Abstract

Cystic hepatic metastasis of squamous cell carcinoma usually develops from necrosis due to insufficient blood supply, yet metastasis initially resembling simple liver cyst is rare. Here, we present a case of a patient with squamous cell lung carcinoma history who found an isolated cystic mass in the liver. Historical MR studies indicated that the mass did not exist 12 months ago and emerged as a small cystic lesion 7 months ago. Radiological findings and tumor markers level suggested metastasis, while 18F-Fluorodeoxyglucose (^18^F-FDG) PET/CT showed moderate tracer uptakes in solid parts of the mass. Pathological study after surgery confirmed metastatic squamous cell carcinoma. Chemotherapy plus recombinant human endostatin and sintilimab therapy was employed after surgery; however, the patient developed remote metastasis of osteolytic lesions in the humerus bone and thoracic vertebra. Our case indicates that metastasis should be taken into consideration in emerging cystic hepatic lesion with malignant history.

## Introduction

The diagnosis of hepatic cystic lesions ranges from benign to malignant situation, leading to different pathogenesis, clinical presentation, and radiological findings (1). Malignant cystic lesions of the liver usually develop from primary hepatobiliary tumor or metastases. The liver acts as a major organ of detoxication and receives blood from most of the digestive organs *via* the hepatic portal vein; thus, the primary source of a hepatic metastasis varies greatly (2, 3), including colon cancer, gastrointestinal stromal tumor (GIST), pancreatic mucinous cystadenocarcinoma, pancreatic neuroendocrine tumor, ovarian cystadenocarcinoma, squamous cell lung cancer, sarcomas, and melanoma. Most of all hepatic metastases arise from the primary cancer of digestive organs. The cystic hepatic metastasis with the original source of squamous cell lung cancer is rare.

The internal cystic portion of hepatic cystic metastasis develops from central necrosis when the tumor outgrows its blood supply (4). The clinical history of primary malignance, epidemic area exposure, or infectious manifestation offers help for its differential diagnosis, which is commonly regarded as hepatobiliary tumor, parasitic disease, infectious abscess, or metastasis. Imaging examinations like contrast-enhanced computed tomography (CT), magnetic resonance (MR), and ultrasound (US) are indispensable methods to differentiate these diagnoses (5, 6). 18F-Fluorodeoxyglucose (^18^F-FDG) PET/CT has been applied as a useful tool for malignance staging due to its whole-body scan and glucose-metabolic assessment for metastases (7). However, it is still necessary to perform aspiration biopsy for definitive pathological diagnosis of hepatic cystic lesion (8). The biopsy sample indicates not only its pathological type but also its gene mutation situation, which is an important referent condition for target therapy and immunotherapy. Target therapy has been realized as a promising strategy for cancer treatment while lots of new target medicine have been applied in clinical practice (9). However, target gene mutation is the prerequisite for its effective response. Immunotherapy has been an efficacious therapeutic approach for hemopoietic system cancer, but the curative response is still unsatisfactory for solid tumor (10).

Here, we present a case of a patient with squamous cell lung carcinoma history who found an emerging isolated cystic mass in the liver. Radiological findings, tumor markers level, and ^18^F-FDG PET/CT suggested metastasis, which was confirmed by pathological study after surgery. Despite of the combined treatment of chemotherapy, anti-angiogenic approach, and programmed cell death protein 1 (PD-1) blockage, the patient developed remote metastasis. Our case indicates that metastasis should be taken into consideration in emerging cystic hepatic lesion with malignant history.

## Case presentation

**Figure 1:**
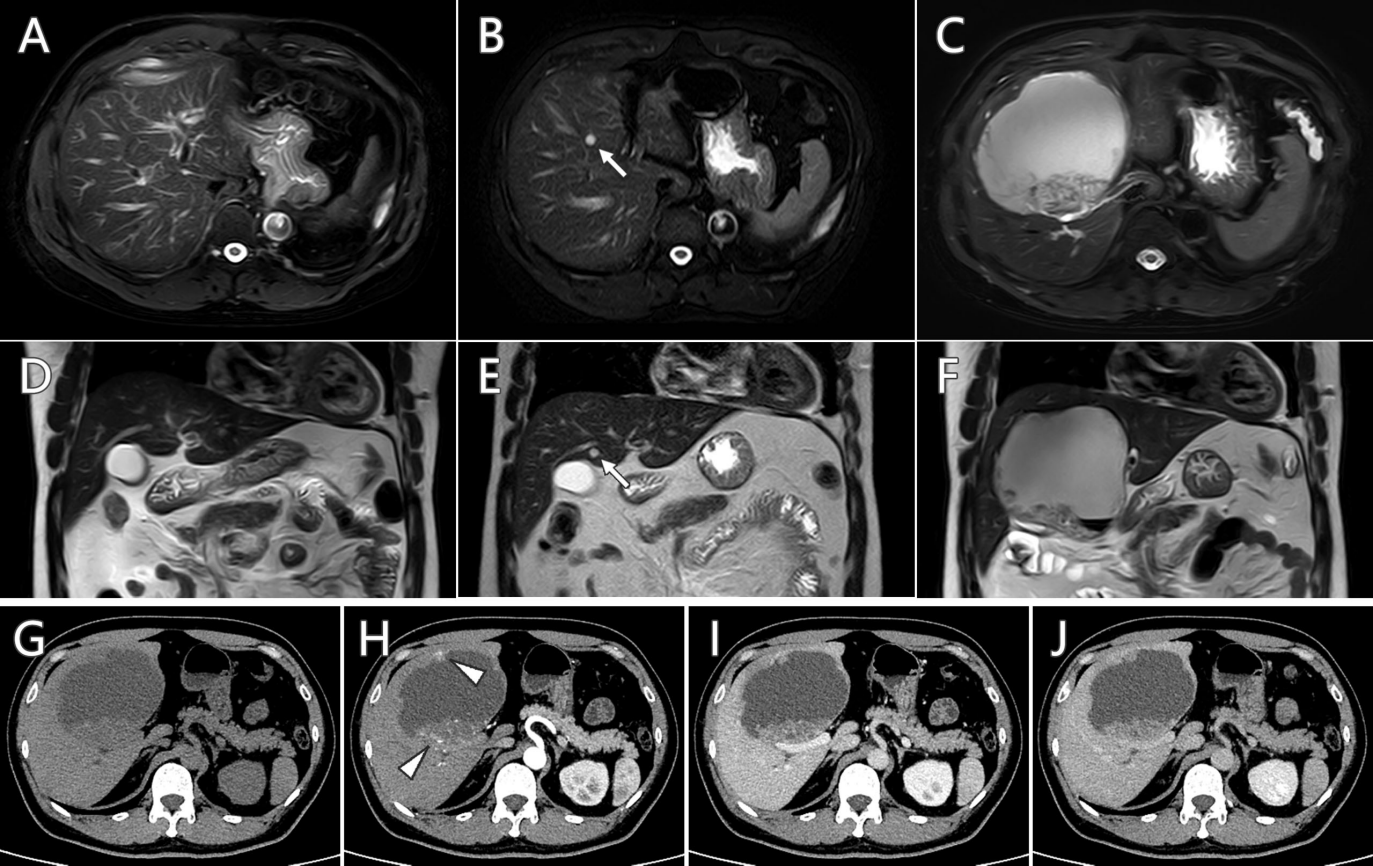
Serial follow-up MR imaging **(A–F)** and contrast-enhanced CT scan **(G–J)** of the hepatic cystic lesion. The newly detected lesion **(C, F)** did not exist 12 months ago **(A, D)** and emerged as a small cystic lesion 7 months ago (**B, E**, arrow). Mild enhancement at the solid parts (arrowhead) was observed in contrast-enhanced CT scan.

A 47-year-old man was admitted to the hospital because of a newly detected mass in the liver. The MR scan (**Figures 1C, F** **)** showed the mass as an isolated hepatic cystic lesion with solid component, with an approximate size of 9.6 cm × 10.7 cm × 9.2 cm. The solid part demonstrated heterogeneous intensity in T1-weighted imaging (T1WI) and T2-weighted imaging (T2WI). The MR scan showed hyperintensity in diffusion-weighted imaging (DWI) and hypointensity in apparent diffusion coefficient (ADC) map at the corresponding area. Historical MR studies indicated that the mass did not exist 12 months ago (**Figures 1A, D**) and emerged as a small cystic lesion 7 months ago (**Figures 1B, E**). Contrast-enhanced CT (**Figures 1G–J**) demonstrated mild enhancement at the solid parts of the mass. Serum tumor marker examination showed that the markers of CEA, CA724, CA199, and ProGRP ascended above normal level, while the markers of CA125, CA153, SCC-Ag, and NSE were at normal level. The liver enzyme of alanine aminotransferase, aspartate aminotransferase, and γ-glutamyl transferase were elevated as well. In comprehensive consideration of radiological findings and tumor markers level, it suggested malignance.

The patient’s medical history stated that he had tumorectomy in the left lung 4 years ago. The histopathological study of the lung tumor reported poorly differentiated squamous cell carcinoma. The immunohistochemical staining was positive for CK5/6, P63, P40, and CD56 and negative for TTF-1, CK7, NapsinA, ROS-1, C-MET, P53, Syn, and CgA. Ki−67 labeling index (Ki-67 LI) was about 80%. Epidermal growth factor receptor (EGFR) gene mutation examination result was negative. After surgery, he received six courses of chemotherapy (gemcitabine and nedaplatin) and 50 Gy of mediastinum radiotherapy, accompanied with two courses of recombinant human endostatin therapy. After that, serial follow-up examinations of CT, MR, and serum tumor markers showed no sign of recurrence in 4 years.

**Figure 2:**
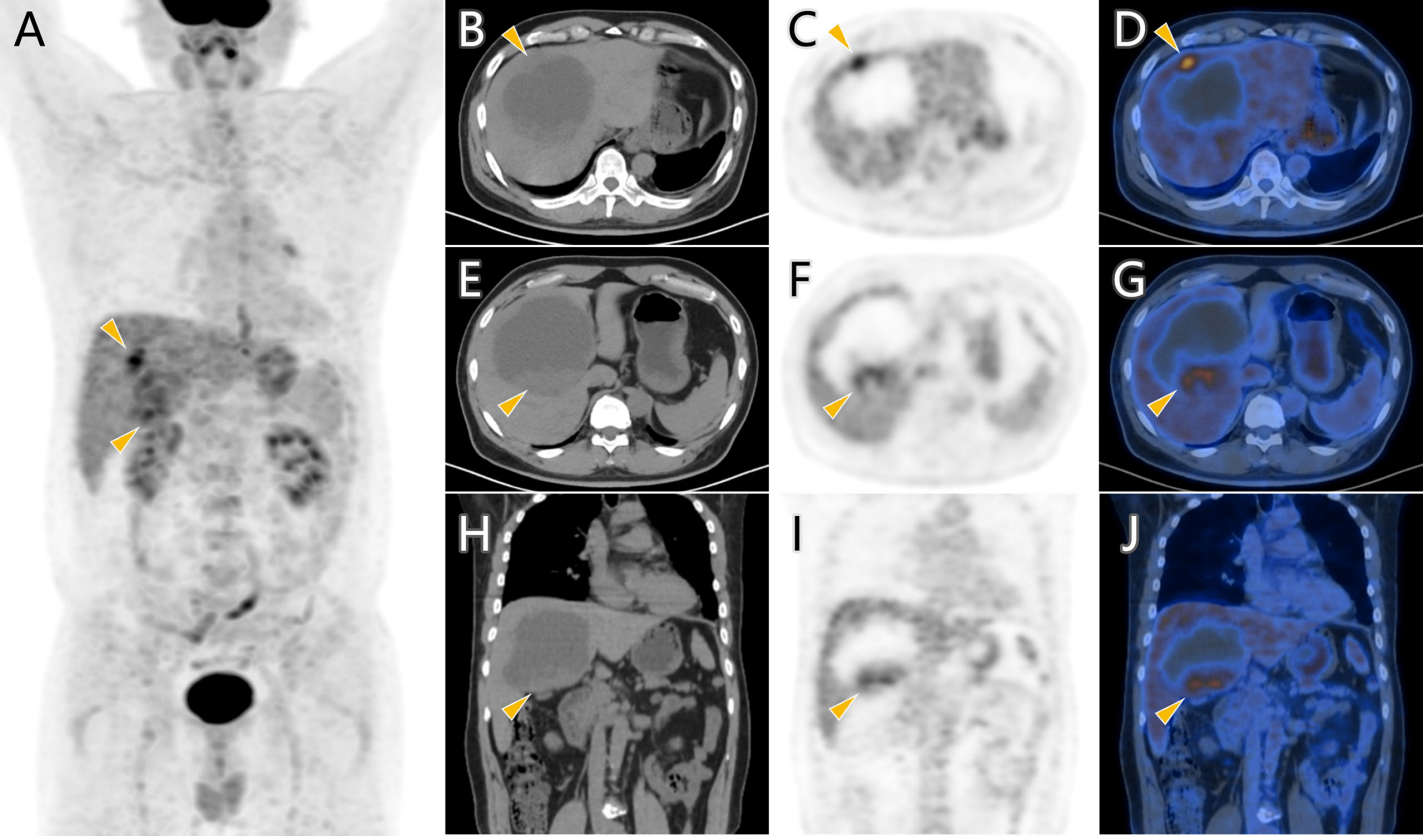
^18^F-FDG PET/CT scan of the hepatic cystic mass. FDG-avid lesions (arrowhead) in MIP image **(A)** were demonstrated as moderate tracer uptakes in solid parts of the mass in axial images **(B–G)** and coronal images **(H–J)**.

**Figure 3:**
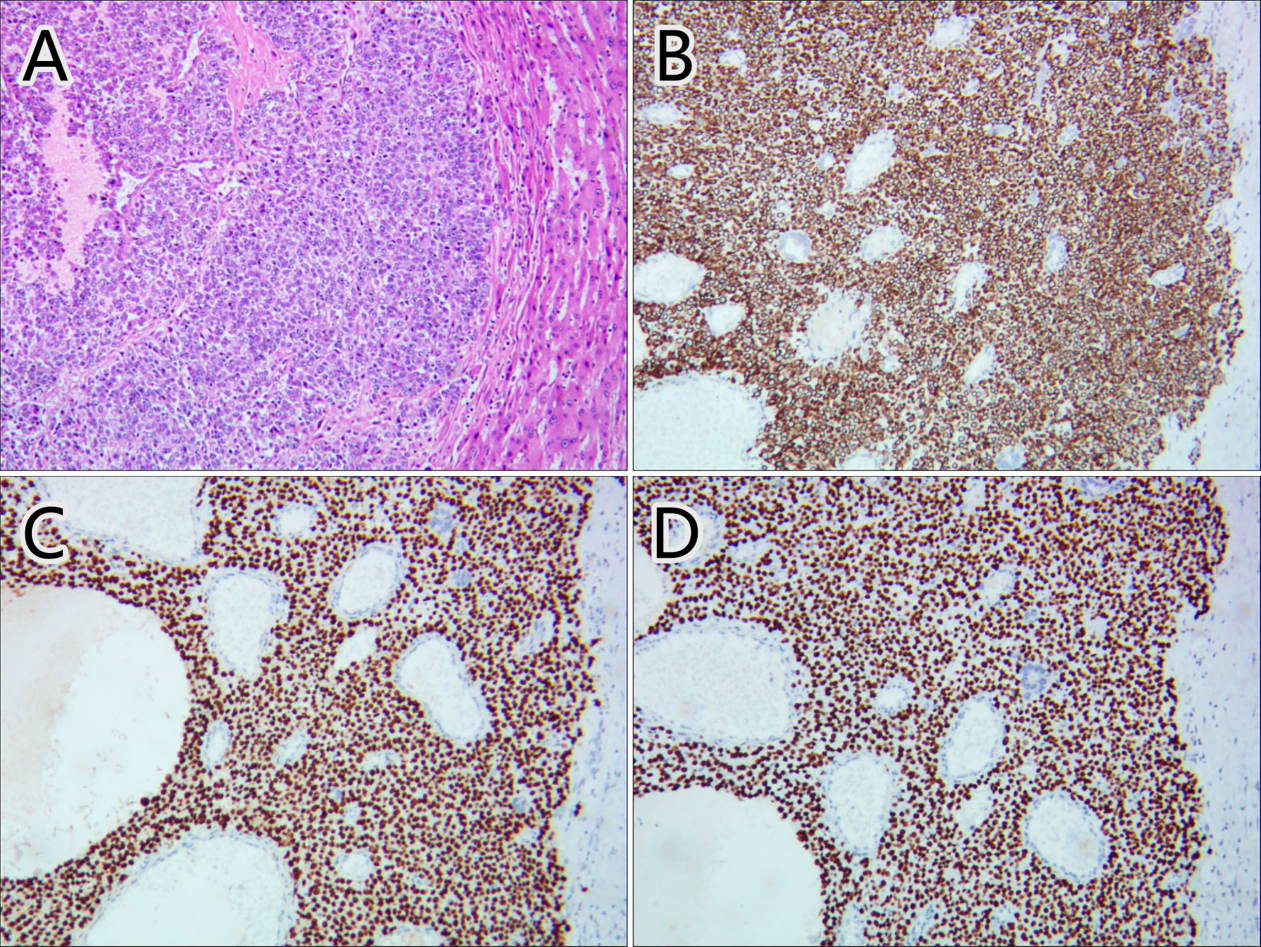
Pathological images of the hepatic mass. Hematoxylin–eosin staining **(A)** and immunohistochemical staining of CK5/6 **(B)**, P63 **(C)**, and P40 **(D)** confirmed the mass as metastatic squamous cell carcinoma.

**Figure 4:**
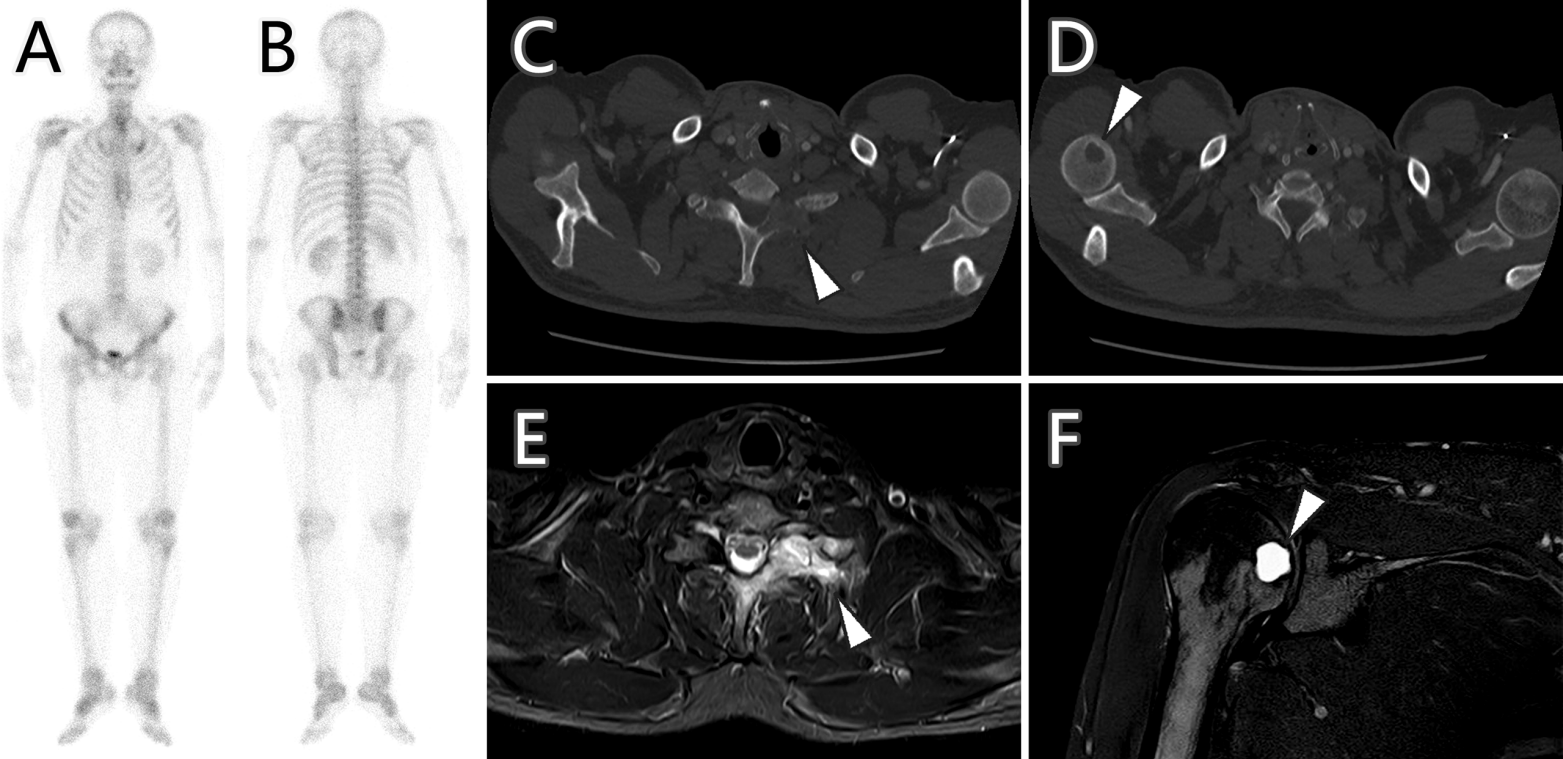
Bone scan, CT imaging, and MR imaging of bone metastases (arrowhead) after 6 months of treatment. Bone scan **(A, B)** showed no increased radioactive uptake, while CT imaging **(C, D)** demonstrated the metastases as osteolytic lesions with hyperintensity in MR spectral attenuated inversion recovery (SPAIR) sequence images **(E, F)**.

Considering the patient’s malignance history, the hepatic mass suggested metastasis. ^18^F-FDG PET/CT was performed for staging (**Figure 2**). Maximum intensity projection (MIP) image showed FDG-avid lesions in the liver, which were demonstrated as moderate tracer uptakes in solid parts of the mass, with SUVmax of 4.8–5.2. Since no other malignant lesions were detected in the PET/CT scan, the patient received tumor resection in November 2021 immediately. The pathological study (**Figure 3**) confirmed metastatic squamous cell carcinoma. The immunohistochemical staining reported positive for CK5/6, P40, P63, CK7, and CK19 and negative for CD56, Syn, CgA, hepatocyte, arginase-1, glypican-3, and CD10. Ki-67 LI was about 30%. Microsatellite instability examination reported stable results. No specific or clinically significant mutation was found for target therapy in lung cancer gene mutation examination, such as ALK, BRAF, EGFR, KRAS, MET, NTRK, PIK3CA, RET, and ROS1. The patient underwent chemotherapy (paclitaxel-albumin and nedaplatin) after surgery, accompanied with recombinant human endostatin therapy and sintilimab therapy. After surgery, the serum tumor markers level of CEA, CA724, and ProGRP declined immediately. However, the tumor marker of CA724 ascended again during chemotherapy (**Supplementary Table S1**), and the patient developed remote metastasis (**Figure 4**). Osteolytic metastatic lesions were found in the humerus bone and thoracic vertebra at the sixth course of chemotherapy. Despite of multiple anti-cancer strategy, it suggested disease progression and poor prognosis.

## Discussion

The common primary sources for cystic hepatic metastasis are colon, kidney, prostate, ovary/testis, squamous cell carcinoma, gastrointestinal stromal tumor (GIST), and neuroendocrine tumor (1). The internal cystic portion may represent central necrosis as the tumor outgrows its blood supply, or mucinous component that is similar to the primary tumor, or secondary cystic degeneration such as GIST after imatinib treatment (2, 11, 12). Imaging examination is able to show the radiological characteristics of the hepatic cystic metastasis, such as size, border definitions, vessel displacement, and dynamic enhancement patterns. Hepatic metastases usually have hyperechoic component along with centrally hypoechoic lesions in US imaging. Cystic metastases are characterized as heterogeneous and ill-defined borders, irregular and incomplete septa, ragged inner surfaces with mural nodules, and enhanced rim in contrast-enhanced imaging examination in CT, MR, and US (13, 14). In our case, the MR and contrast-enhanced CT findings of the cystic metastasis were in accordance with the above-mentioned radiological characteristics. ^18^F-FDG PET/CT has been applied as an effective examination in the field of benign and malignant diagnosis, prognosis evaluation, treatment efficacy evaluation, tumor recurrence detection, searching for an unknown primary with metastasis, biopsy guidance, and pre-surgical planning (15). The ^18^F-FDG PET/CT scan in our case showed the malignance as a solitary mass with no other metastasis, which provided evidence for further surgical management.

The differential diagnoses of the hepatic cystic lesion can be divided into developmental, inflammatory, neoplastic, and trauma-related lesions (6, 16). Liver abscess is an important differential diagnosis for cystic hepatic metastases due to the complex symptoms or the indeterminate imaging features (17). In order to achieve more definitive diagnosis, it is necessary to integrate imaging examination with clinical and laboratory findings. Most of the hepatic cystic metastases share the same features with the primary tumor source. However, the metastasis in our case has its own characteristics. First, the metastasis was an isolated lesion, differing from multifocal lesions. Second, the cystic lesion initially resembled a simple liver cyst with fast growth and evolved into a cystic mass, which was different from solid metastasis developing necrosis due to insufficient blood supply (18). This atypical feature has also been illustrated in another case report of a nasopharyngeal carcinoma (NPC) patient who found liver cystic lesion after chemoradiotherapy, which initially resembled a simple liver cyst with fast growth and evolved into an abscess-like mass (14). Third, the moderate FDG uptake of the cystic lesion also differed from strong tracer uptake of regular primary squamous cell carcinoma in PET/CT scan. This case indicates that it may present dissimilar tumor behavior between original tumor and metastatic lesion. Moreover, the original tumor in this case is a poorly differentiated carcinoma, which may develop more variability and heterogeneity in metastasis than well-differentiated carcinoma (19). The spatial and temporal variability of biomarkers in solid tumors has been reported to explain the discrepancies between primary tumor and metastasis, suggesting the development of personalized medicine in oncology (20).

Despite of its rarity, cystic hepatic metastases of squamous cell carcinoma have been reported in other articles with various primary origins. Here, we present some cases that have exhibited representative characteristics. The first case noted that a large, well-defined, lobulated cystic lesion with poor contrast enhancement occupying both lobes of the liver was detected in a 52-year-old man who had NPC history 4 years ago (21). The biopsy of the hepatic cystic lesion reported poorly differentiated squamous cell carcinoma, and *in situ* hybridization for Epstein–Barr virus early RNAs confirmed the diagnosis of metastatic NPC. The second case was a 38-year-old female patient who had been diagnosed with cervical squamous cell carcinoma 10 months ago (22). The PET/CT scan found a cystic lesion in the liver after radiotherapy, which was diagnosed as squamous cell carcinoma metastasis by aspiration biopsy. The third case was a 69-year-old woman who had anal squamous cell carcinoma and underwent chemoradiotherapy and salvage abdominoperineal resection (23). She developed multifocal cystic lesions in the liver after surgery, and the lesions were revealed as metastatic squamous cell carcinoma by cytology of liver drainage and liver biopsy. All the above cases indicate that the hepatic metastasis of squamous cell carcinoma may have pseudocystic presentation; therefore, biopsy is necessary to manage definitive diagnosis.

Target therapy and immunotherapy have been realized as effective strategies for cancer management. These strategies use small pharmacological agent or monoclonal antibody in cancer lesions to prevent growth signal, stop angiogenesis signal, inhibit hormone supply, trigger cell death, or assist immune system recognition (24), which demonstrate dramatic therapeutic response. Our case showed no specific mutation in lung cancer gene mutation examination; thus, he could not benefit from specific target therapy against EGFR, ALK, BRAF, etc. Endostatin has the activity as modifiers of both angiogenesis and endothelial cell autophagy (25). A meta-analysis has investigated recombinant human endostatin combined with chemotherapy in patients with squamous cell lung cancer, which indicated better therapeutic effect by the combined treatment than chemotherapy alone, with no increased incidence of adverse reactions (26). In our case, the patient was tumor free for 44 months after the primary lung carcinoma treatment with endostatin and chemoradiotherapy. As an anti-PD-1 monoclonal antibody, sintilimab has been approved for squamous and non-squamous lung carcinoma in China, and the combination of sintilimab with other anti-cancer strategies have shown promising therapeutic efficacy (27). In a phase 3 clinical trial study of sintilimab plus platinum and gemcitabine as the first-line treatment for advanced or metastatic squamous non-small cell lung cancer, the combined treatment revealed better progression-free survival than platinum and gemcitabine treatment (28). However, in our case, the endostatin and sintilimab treatment did not prevent remote metastasis after the secondary hepatic metastasis therapy, suggesting poor prognosis.

In conclusion, our case indicates that metastasis should be taken into consideration when emerging cystic lesion is observed in the liver, especially in a situation with malignant history.

## Data availability statement

The original contributions presented in the study are included in the article/**Supplementary Material**. Further inquiries can be directed to the corresponding author.

## Author contributions

CL contributed to data acquisition and analysis, literature review, and manuscript drafting and revision. XC and HS acquired examination image and interpreted data. LX collected the patient’s necessary information (including the laboratory test results, the examination results, and the pathological reports). DL supervised the information collection and revised the manuscript. All authors contributed to the article and approved the submitted version.

## Conflict of interest

The authors declare that the research was conducted in the absence of any commercial or financial relationships that could be construed as a potential conflict of interest.

## Publisher’s note

All claims expressed in this article are solely those of the authors and do not necessarily represent those of their affiliated organizations, or those of the publisher, the editors and the reviewers. Any product that may be evaluated in this article, or claim that may be made by its manufacturer, is not guaranteed or endorsed by the publisher.
