# Supplementary material for: Case report: Rare isolated cystic hepatic metastasis of a patient with squamous cell lung carcinoma history and the prognosis
Source: Front Oncol. 2022 Oct 27;12:986603. doi: 10.3389/fonc.2022.986603 (PMC9648660; doi:10.3389/fonc.2022.986603)
Supplement: Supplementary file 1 [file Table_1.docx]

**TABLE 1.** Serial tumor markers level after the hepatic metastasis resection.

|  | Nov, 2021 | Jan, 2022 | Jan, 2022 | Feb, 2022 | Mar, 2022 | Mar, 2022 | Apr, 2022 | May, 2022 |
| --- | --- | --- | --- | --- | --- | --- | --- | --- |
| SCC-Ag  (0-1.5ng/ml) | 1 | 0.8 | 0.6 | 0.6 | 0.5 | 0.3 | 0.5 | 0.2 |
| ProGRP  (0-70pg/ml) | 93 | 16.71 | 23.24 | 22.86 | 19.9 | 25.52 | 22.23 | 26.2 |
| Cyfra 21-1  (0-2.08ng/ml) | - | 1.1 | 0.89 | 1.12 | - | 1.13 | 0.76 | 0.71 |
| CA125  (0-35U/mL) | 16.1 | 34.1 | 14.7 | 11.7 | 11.7 | - | - | - |
| CEA  (0-5ng/ml) | 6.63 | 0.55 | 0.53 | 0.94 | 0.89 | 0.76 | 1 | 0.87 |
| CA724  (0-6.9IU/mL) | 52.24 | 10.36 | 68.29 | 141.6 | 105.2 | - | - | - |
| NSE  (0-16.3ng/ml) | 8.58 | 8.72 | 10.39 | 10.06 | 8.21 | 7.85 | 9.24 | 7.12 |
